# uL3 Mediated Nucleolar Stress Pathway as a New Mechanism of Action of Antiproliferative G-quadruplex TBA Derivatives in Colon Cancer Cells

**DOI:** 10.3390/biom10040583

**Published:** 2020-04-10

**Authors:** Annalisa Pecoraro, Antonella Virgilio, Veronica Esposito, Aldo Galeone, Giulia Russo, Annapina Russo

**Affiliations:** Department of Pharmacy, University of Naples “Federico II”, Via Domenico Montesano 49, 80131 Naples, Italy; annalisa.pecoraro@unina.it (A.P.); antonella.virgilio@unina.it (A.V.); veronica.esposito@unina.it (V.E.); aldo.galeone@unina.it (A.G.)

**Keywords:** G-quadruplex aptamers, TBA derivatives, nucleolar stress, ribosomal protein uL3, cancer therapy

## Abstract

The antiproliferative G-quadruplex aptamers are a promising and challenging subject in the framework of the anticancer therapeutic oligonucleotides research field. Although several antiproliferative G-quadruplex aptamers have been identified and proven to be effective on different cancer cell lines, their mechanism of action is still unexplored. We have recently described the antiproliferative activity of a heterochiral thrombin binding aptamer (TBA) derivative, namely, LQ1. Here, we investigate the molecular mechanisms of LQ1 activity and the structural and antiproliferative properties of two further TBA derivatives, differing from LQ1 only by the small loop base-compositions. We demonstrate that in p53 deleted colon cancer cells, LQ1 causes nucleolar stress, impairs ribosomal RNA processing, leading to the accumulation of pre-ribosomal RNAs, arrests cells in the G2/M phase and induces early apoptosis. Importantly, the depletion of uL3 abrogates all these effects, indicating that uL3 is a crucial player in the mechanism of action of LQ1. Taken together, our findings identify p53-independent and uL3-dependent nucleolar stress as a novel stress response pathway activated by a specific G-quadruplex TBA derivative. To the best of our knowledge, this investigation reveals, for the first time, the involvement of the nucleolar stress pathway in the mechanism of action of antiproliferative G-quadruplex aptamers.

## 1. Introduction

Aptamers are relatively short DNA and RNA oligonucleotides endowed with high affinity and specificity toward a given target molecule [1]. Although not exclusively, aptamers are usually discovered by technology, or variants of it, called SELEX [2]. Shortly, by starting from random oligonucleotide sequences and through selection and amplification steps, this method allows the identification of ligands able to adopt peculiar tridimensional structures. Thanks to their outstanding properties, aptamers can be regarded as promising therapeutic and/or diagnostic tools, which are often considered a valid alternative to antibodies in several applications.

One of the crucial features of aptamers during the SELEX process is their ability to fold in properly stable nucleic acid secondary structures. For example, typical structural motives occurring in aptamers include hairpins, pseudoknots, and loops. However, not surprisingly, a considerable number of aptamers with significant biological activities are characterized by G-rich sequences, thus adopting G-quadruplex structures such as scaffolds (G4-aptamers), which are among the most stable nucleic acid conformations [3]. The core unit of a G-quadruplex is a planar squared arrangement of four guanines (G-tetrad) interconnected by eight Hoogsteen hydrogen bonds. Two or more G-tetrads can stack on each other, generating the G-quadruplex structure, which is further stabilized by cations accommodated in its cavity. The outstanding polymorphism of the G-quadruplex structures is the key feature that grants to the G4-aptamers the ability to interact with several different targets.

Although the G4-aptamers investigated up to now have a large potential in therapeutics, diagnostics, and analytics, in terms of drug development, the most promising results have been obtained in the research field of antiproliferative and anticancer agents. However, despite the evident therapeutic potential of antiproliferative G-quadruplex forming ODNs, the research in this field is considerably challenging for some features, in particular, (1) the high extent of polymorphism shown by many antiproliferative G-rich sequences [4,5], (2) the contribution of guanine-based degradation products to the antiproliferative activity [6], (3) the recently described multi-targeted effects of antiproliferative G4-aptamers [7], and (4) the multitude of molecular pathways potentially involved in the antiproliferative activity [1].

**Figure 1:**
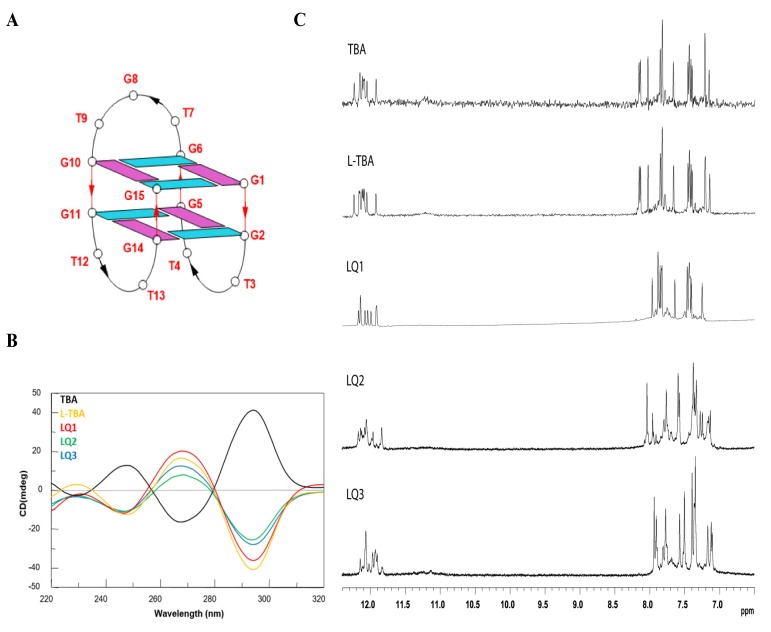
Structural investigations of the thrombin binding aptamer (TBA) derivatives. (**A**) Schematic representation of the G-quadruplex structure adopted by TBA. Guanosines in *syn* and *anti* glycosidic conformations are in purple and light blue, respectively. (**B**) CD spectra at 20 °C of the modified TBAs and their natural counterpart at 50 µM ODN strand concentration in a buffer solution 10 mM KH_2_PO_4_*/*K_2_HPO_4_, 70 mM KCl (pH 7.0). (**C**) Aromatic and imino proton regions of the ^1^H NMR spectra (500 MHz) of the ODNs investigated (Table 1). See the Materials and Methods section for details.

In the area of interest of G4-aptamers, the antiproliferative activity of TBA, formerly developed as an anticoagulant agent, was first described in 2003 [8]. This aptamer folds in a well-defined antiparallel chair-like right-handed G-quadruplex structure characterized by two stacked G-tetrads connected through three loops, specifically two small TT and one large TGT loops (Figure 1A) [9]. Nonetheless, the exploitation of TBA as an antiproliferative agent has been stalled by its concomitant anticoagulant activity and low resistance to enzymes in physiological conditions.

Recently, many of the investigations in this field have been devoted to derivatives of TBA containing backbone modifications [10,11,12,13,14] or non-canonical bases [15,16,17], with the aim to preserve the antiproliferative activity to the detriment of the anticoagulant activity and improve the resistance to nucleases [14], in view of their development as potential anticancer agents.

The pathogenesis of cancer is very complicated, and finding novel anti-tumor drugs with high selectivity and few side effects is still one of the main aims of the anti-tumor drug research. Several findings have demonstrated that ribosome biogenesis is consistently hyperactivated in cancer [18]. To provide optimal efficiency of protein biosynthesis, nucleoli must supply a great number of ribosomes. Despite this key function in the maintenance of cell homeostasis, a growing number of data show that nucleoli function as stress sensors, modulating the activities of different oncogenes and tumor suppressors involved in DNA repair, cell cycle progression, cell growth, cell proliferation, and senescence [19]. Ribosomal proteins (r-proteins) are nucleolar proteins that, in addition to their role in ribosome biogenesis, show extra-ribosomal functions, some of which affect cancer biology [20,21]. uL3, formerly named rpL3, belongs to this subset of r-proteins, and, as extra-ribosomal player, is involved in a number of cellular events activated in response to cell stressors that trigger the so-called “nucleolar stress response” [21,22,23,24]. The expression levels of uL3 require specific regulatory strategies, including the association of alternative splicing and nonsense-mediated mRNA decay (AS-NMD) [25]. The activity of ribosome free uL3 has been intensively investigated by some of us. A key role of ribosome-free uL3 is to arrest cell cycle progression and/or to induce apoptosis in response to different conventionally used chemotherapeutic drugs, interfering with ribosome biogenesis, such as Actinomycin D, 5-fluorouracile, Oxaliplatin, and Niclosamide [21,22,24,25,26,27].

In 2017, we described the antiproliferative activity of several TBA derivatives against two different cancer cell lines, namely Calu-6 and HCT 116^p53−/−^ [11]. Two of these previously investigated TBA derivatives attracted our attention, specifically L-TBA, composed exclusively by L-residues, and LQ1 (formerly D13), composed by L-nucleosides except for those ones in the small loops (Table 1), considering that they possess no anticoagulant activity and have shown exceptional resistance to nucleases [28].

The aim of the present paper is the synthesis of new G-quadruplex aptamer TBA derivatives and the analysis of their antiproliferative activity. In addition, we investigate the molecular mechanism underlying the cytotoxic activity of L-TBA and LQ1 [11]. We demonstrate that, in p53-deleted colon cancer cells, the cytotoxic activity of LQ1 is mediated by the ribosomal protein uL3 and is associated to the activation of a p53-independent nucleolar stress pathway. Moreover, we investigate the structural and antiproliferative properties of two further TBA derivatives differing from LQ1 only by the D/L-residues composition of the small loops (namely LQ2 and LQ3) and specifically designed to maintain thymidines T4 and T13, important for structural stability, with the same chirality.

## 2. Materials and Methods

### 2.1. Oligonucleotides Synthesis and Purification

Modified ODNs were synthesized on a Millipore Cyclone Plus DNA synthesizer using solid-phase β-cyanoethyl phosphoramidite chemistry at a 1-µmol scale. The synthesis was performed by using in addition to normal 3′-phosphoramidites, modified phosphoramidite monomers, such as 5′-dimethoxytrityl-β-L-deoxyguanosine(iBu)-3′-phosphoramidite and 5′-dimethoxytrityl-β-L-deoxythymidine-3′-phosphoramidite, commercially available (ChemGenes, Wilmington, MA, USA). For all ODNs, an universal support was used. The oligomers were detached from the support and deprotected by treatment with concentrated aqueous ammonia at 80 °C overnight. The combined filtrates and washings were concentrated under reduced pressure, redissolved in H_2_O, analyzed and purified by high-performance liquid chromatography on a Nucleogel SAX column (Macherey-Nagel, 1000-8/46, Macherey-Nagel, Düren, Germany), using buffer A: 20 mM KH_2_PO_4_/K_2_HPO_4_ aqueous solution (pH 7.0) containing 20% (*v/v*) CH_3_CN and buffer B: 1 M KCl, 20 mM KH_2_PO_4_/K_2_HPO_4_ aqueous solution (pH 7.0) containing 20% (*v/v*) CH_3_CN; a linear gradient from 0 to 100% B for 45 min and flow rate of 1 ml/min were used. The fractions of the oligomers were collected and successively desalted by Sep-pak cartridges (C-18). The isolated oligomers proved to be > 98% pure by NMR.

### 2.2. NMR Spectroscopy

NMR samples were prepared at a concentration of about 3.0 mM in 0.6 mL (H_2_O/D_2_O 9:1 *v/v*) buffer solution having 10 mM KH_2_PO_4_/K_2_HPO_4_, 70 mM KCl, and 0.2 mM EDTA (pH 7.0). All the samples were heated for 5–10 min at 90 °C and slowly cooled (10–12 h) to room temperature. The solutions were equilibrated for several days at 4 °C. The annealing process was assumed to be complete when 1H NMR spectra were superimposable on changing time. NMR spectra were recorded with a Varian Unity INOVA 500 MHz spectrometer. 1D proton spectra of the sample in H_2_O were recorded using pulsed-field gradient DPFGSE for H_2_O suppression. 1H-chemical shifts were referenced relative to external sodium 2,2-dimethyl-2-silapentane-5-sulfonate (DSS). Pulsed-field gradient DPFGSE sequence was used for NOESY (180 and 80 ms mixing times) and TOCSY (120 ms mixing time) experiments in H_2_O. All experiments were recorded using the STATES-TPPI procedure for quadrature detection. In all 2D experiments, the time domain data consisted of 2048 complex points in t2 and 400–512 fids in t1 dimension. A relaxation delay of 1.2 s was used for all experiments.

### 2.3. CD Spectroscopy

CD samples were prepared at a concentration of 50 µM by using a buffer solution: 10 mM KH_2_PO_4_*/*K_2_HPO_4_, 70 mM KCl, pH 7.0. CD data of all quadruplexes were registered on a Jasco 715 CD spectrophotometer in a 0.1-cm pathlength cuvette. For the CD spectra, the wavelength was varied from 220 to 320 nm at a 100 nm min^−1^ scan rate, and the spectra recorded with a response of 16 s at 2.0 nm bandwidth and normalized by subtraction of the background scan with buffer. The temperature was kept constant at 20 °C with a thermoelectrically-controlled cell holder (Jasco PTC-348, Jasco Europe s.r.l., Cremella (LC), Italy). CD melting and annealing curves were registered as a function of temperature from 20 to 90 °C at 294 nm with a scan rate of 30 °C/h.

### 2.4. Cell Cultures and Treatments with the ODNs

HCT 116^p53−/−^ (American Type Culture Collection, (ATTC) Manassas, Virginia, USA), uL3∆HCT 116^p53−/−^, derived from HCT 116^p53−/−^ cell line and stably silenced of uL3 [29] and eL8∆HCT 116^p53−/−^ cells were cultured in Dulbecco’s modified Eagle’s medium (DMEM), supplemented with 10% fetal bovine serum (FBS), 2 mM L-glutamine and 50 U/ml penicillin-streptomycin, under a humidified atmosphere of 5% CO_2_ at 37 °C.

eL8∆HCT 116^p53−/−^ cells were derived from an HCT 116^p53−/−^ cell line and stably silenced of eL8 by using specific shRNA (Santa Cruz, Dallas, TX, USA). shRNA transfections were performed in cells, as previously described [25].

Treatments of cells were performed replacing the culture medium with those containing different ODNs at a final concentration of 10 and 50 μM per well from 24 to 72 h.

### 2.5. Western Blotting Analysis

Protein extracts were prepared as previously described [30]. Western blotting analysis was performed as previously reported [31]. The membranes were challenged with anti-uL3, anti-eL8 (Primm, Milan, Italy), anti-uL5, anti-uL18 (Cell Signaling Technology, Danvers, MA, USA), anti-Bax, anti-p21, anti-CycB1, anti-α-tubulin, anti-α-actin, anti-GAPDH, and anti-Vinculin (Santa Cruz, Dallas, TX, USA). Proteins were visualized with enhanced chemiluminescence detection reagent according to the manufacturer’s instructions (Elabscience®, Houston, TX, USA).

### 2.6. MTT Assay

HCT 116^p53−/−^ cells were seeded onto 96-well plates at a density of 1 × 10^4^ cells/well and treated with different ODNs at a final concentration of 10 and 50 μM from 24 h to 72 h. Then, cell viability was determined using the MTT assay, as previously reported [11]. A pool of three different sets of experiments was performed. Error bars represent mean ± SEM from *n* = 3 biological replicates.

### 2.7. RT-qPCR

Total RNA was isolated from cells, as previously described [32]. RNA was retrotranscribed using a SensiFAST^TM^ cDNA Synthesis kit (Bioline, London, UK), and then real-time PCR was carried out using a SensiFAST SYBER® No-ROX kit (Bioline, London, UK). The primers are indicated in Table 2. The comparative Ct method was used to calculate the relative abundance of the mRNA and compared with that of β-actin expression [33].

### 2.8. Cell Cycle Analysis by Flow Cytometry

HCT 116^p53−/−^ cells, uL3ΔHCT 116^p53−/−^ and eL8∆HCT 116^p53−/−^ cells were seeded into 60 mm tissue culture plates at confluency of about 50%–60%. Then, cells were treated with LQ1 at 10 μM. After 48 h, the cells (2 × 10^6^) were harvested and centrifuged at 400× *g* for 5 min, washed once with cold PBS (Dulbecco’s phosphate-buffered saline, Sigma-Aldrich, St. Louis, MO, USA) and resuspended and fixed by adding 0.5 ml ice-cold 70% ethanol dropwise. Then, the cells were incubated on ice overnight. The cells were spun down and washed twice with PBS. They were resuspended in 500 μl of PBS and incubated with 200 μg/mL RNAse (Sigma-Aldrich, St. Louis, MO, USA) for 30 min at 37 °C; then, 50 μg/mL PI (Propidium Iodide, Sigma-Aldrich, St. Louis, MO, USA) was added and cell lysate was incubated for 30 min protected from light. Cell cycle distribution was analyzed using BD FACS CantoII Cytometer (BD Biosciences, San Jose, CA, USA) and Diva software (v6.x).

### 2.9. Cell Death Assay

HCT 116^p53−/−^ cells and uL3ΔHCT 116^p53−/−^ cells were seeded into 60 mm tissue culture plates at a confluency of about 50%–60%. Then, cells were treated with LQ1 at 10 μM. After 48 h, the cells were stained using the Tali^®^ Apoptosis Kit, Annexin V Alexa Fluor^®^ 488, and Propidium Iodide (PI; Life Technologies, Carlsbad, CA, USA). Briefly, cells were harvested, stained with Annexin V Alexa Fluor^®^ 488 in the dark for 20 min at room temperature, and then stained with PI for 5 min. Cell apoptosis was analyzed using BD FACS CantoII Cytometer (BD Biosciences, San Jose, CA, USA) and Diva software (v6.x).

### 2.10. Statistical Analysis

Statistical analysis was performed as previously reported [34].

## 3. Results

### 3.1. Structural Insight of the Investigated Sequences

Circular dichroism (CD) is an excellent tool for rapid determination of the occurrence of G-quadruplex structures in solution and to obtain preliminary information about their folding properties. One of the most straightforward applications of CD to G-quadruplex investigation is to determine whether a sequence analogue of a parent ODN is similarly folded, or if a chemical modification is able to affect its conformation or stability. In Figure 1B, the CD profiles of the ODNs investigated are shown together with that of the natural TBA, that shows the typical profile of an antiparallel G-quadruplex in which *anti* and *syn* guanosines alternate along the strands, being characterized by two positive bands at 247 and 295 nm, and a negative one at 266 nm.

As previously reported [28], L-TBA shows a CD profile completely specular to that of its unmodified analog, thus indicating that this ODN adopts a left-handed chair-like G-quadruplex structure that is the mirror image of the TBA structure, as expected. Although the sequences of the other modified differ from L-TBA in the D/L-residues composition of the loop (Table 1), their CD spectra show profiles very similar to that of L-TBA, except for slight differences in the band intensities more marked for LQ2 and LQ3. These data are indicative of the occurrence of a left-handed chair-like G-quadruplex structure for all the modified sequences. These results are not particularly surprising, taking into consideration that the core of the G-quadruplex structure, mostly affecting the helix handedness, is only composed of L-residues.

The CD heating/cooling curves can provide further information about the G-quadruplex structure adopted by modified ODNs and, in particular, they are used to determine the effects of chemical modifications on G-quadruplex thermal stability. The estimated melting temperatures (T_m_) are listed in Table 1. A comparison of data here and previously reported [28] shows that T_m_ of all modified analogs is quite similar to that of the parent aptamer. Noticeably, LQ1 exhibits a slightly higher thermal stability (52 °C) than the unmodified TBA (Table 1).

Similar to the parent TBA and other TBA derivatives, CD melting/annealing profiles of LQ2 and LQ3 are almost superimposable (Figure S1), thus being consistent with the presence of monomolecular G-quadruplex structures for these modified aptamers, as already suggested by the CD profiles and clearly indicated by the NMR characterization.

The ability of all analogs to fold into a TBA-like antiparallel G-quadruplex was also assessed by NMR spectroscopy. The simple appearance of all ^1^H NMR spectra indicates that, in the conditions used here, the modified ODNs form mainly a single well-defined hydrogen-bonded conformation, such as for TBA. In fact, their one-dimensional ^1^H NMR spectra (500 MHz, T = 25 °C; Figure 1C), in the K^+^ containing buffer utilized, show, principally, the presence of eight signals in the region 11.7–12.4 ppm, attributable to imino protons involved in Hoogsteen hydrogen bonds of at least two G-quartets and fifteen main signals in the aromatic region (7.0–8.5 ppm), due to the presence of nine guanine H8 and six thymine H6 protons, clearly indicating the formation of a well-defined G-quadruplex structure, as in the case of the parent aptamer. In all cases, more detailed 2D-NMR investigations confirmed a monomolecular chair-like G-quadruplex structure similar to that of the original TBA, obviously except for the helix type. A combination of the analysis of 2D NOESY (Figures S2 and S3) and TOCSY spectra (data not shown) allowed us to obtain the almost complete assignment (Table S1) of the nonexchangeable protons for LQ2 and LQ3.

### 3.2. Cytotoxicity Activity of Investigated ODNs

**Figure 2:**
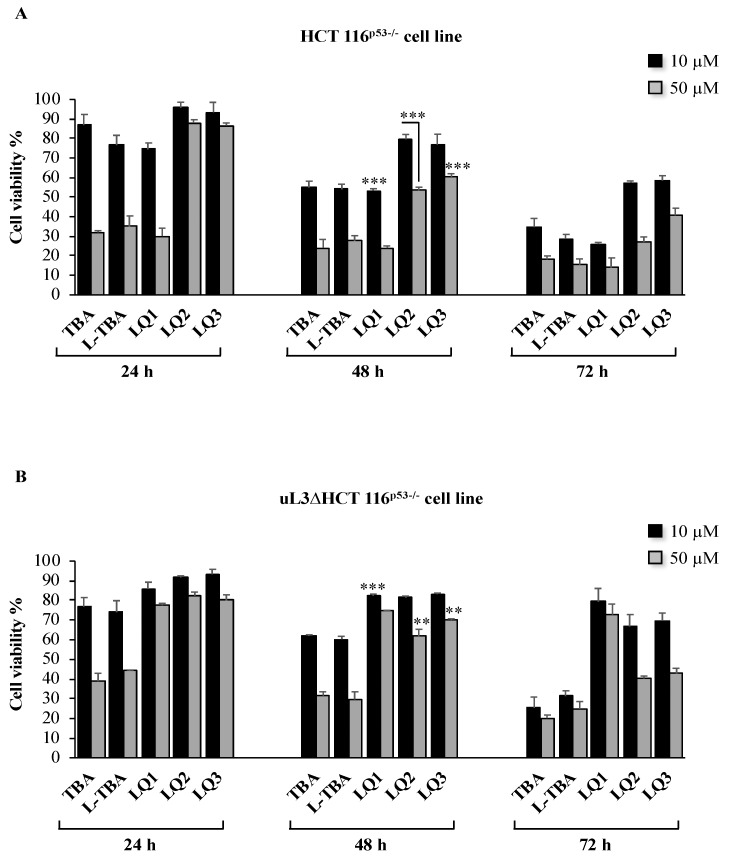
Cytotoxic activity of L-TBA and its derivatives on HCT 116^p53−/−^ (**A**) and uL3ΔHCT 116^p53−/−^ (**B**) cells. Cells have been treated with 10 and 50 μM of ODNs from 24 to 72 h. Cell viability was assayed using the MTT assay. Results are presented as percentage of the untreated cells. Bars represent the mean of triplicate experiments; error bars represent the standard deviation. ** *p* < 0.01, *** *p* < 0.001 vs. untreated cells set at 100%.

To evaluate the cytotoxic activity of LQ2 and LQ3, HCT 116^p53−/−^ cells were incubated with 10 and 50 μM of these ODNs and cell cytotoxicity was tested at 24, 48, and 72 h of treatment. Results of MTT assay were compared to those obtained after the treatment of cells with TBA, L-TBA, and LQ1 (already D13) recently published [11], in the same conditions. Figure 2A shows that LQ2 and LQ3 have low cytotoxicity compared to that of the others.ODNs tested at both concentrations and all time points. In particular, 48 h after the treatment at 50 μM, HCT 116^p53−/−^ cells retained about 60% of viability as compared with control (untreated cells set to 100%). The cell viability of HCT 116^p53−/−^ treated with TBA, L-TBA, and LQ1 in the same experimental conditions was about 20%–30%. Next, we tested the cytotoxic activity of all ODNs in uL3∆HCT 116^p53−/−^ cells, a cell subline stably silenced of uL3 derived from HCT 116^p53−/−^ cells [29], uL3 silencing efficiency in these cells is shown in Figure S4. To this aim, uL3∆HCT 116^p53−/−^ cells were exposed to 10 and 50 μM of all ODNs from 24 to 72 h. Then, cytotoxicity was evaluated by MTT assay. Figure 2B shows that in uL3∆HCT 116^p53−/−^ cells, uL3 silencing did not modify the cell response to the treatment with TBA, L-TBA, LQ2, and LQ3 at 10 and 50 μM in all tested time points. In fact, the cells viability was comparable to that of parental cells expressing uL3. These data indicate that the cytotoxicity activity of these ODNs was independent of uL3 status.

Nevertheless, the depletion of uL3 completely abolished the cytotoxicity of LQ1. In fact, upon the silencing of uL3, the treatment of cells with LQ1 was associated to a strong increase of cell viability (about 80% vs. control in all tested time points) when compared to the results obtained in the presence of uL3 in the same experimental conditions (Figure 2B).

These data clearly demonstrate that uL3 is essential to mediate cell response to LQ1 treatment in HCT 116 ^p53−/−^ cells.

### 3.3. LQ1 Causes Nucleolar Stress and Affects rRNA Processing

We have previously demonstrated that uL3 is a key player in the activation of a p53-independent nucleolar stress pathway in HCT 116^p53−/−^ cells upon treatment with chemotherapeutic drugs altering ribosome biogenesis [21]. In this context, the results of MTT assays that suggest a role of uL3 in the cytotoxic activity of LQ1 led us to hypothesize that the activity of this ODN could be mediated by a nucleolar stress pathway involving uL3. To test this hypothesis, we analyzed the expression profile of uL3 and a subset of ribosomal proteins known to be involved in the nucleolar stress response, including uL5, uL11, uL18, uS12 [21,35,36], together with the nucleolar marker B23/NPM after treatment of the cells with LQ1. To this aim, HCT 116^p53−/−^ and uL3∆HCT 116^p53−/−^ cells were treated with 10 μM of LQ1, and, 48 h later, the relative abundance of uL3, uL5, uL11, uL18, uS12, and B23/NPM mRNAs was determined by RT–qPCR with specific primers (Table 2).

**Figure 3:**
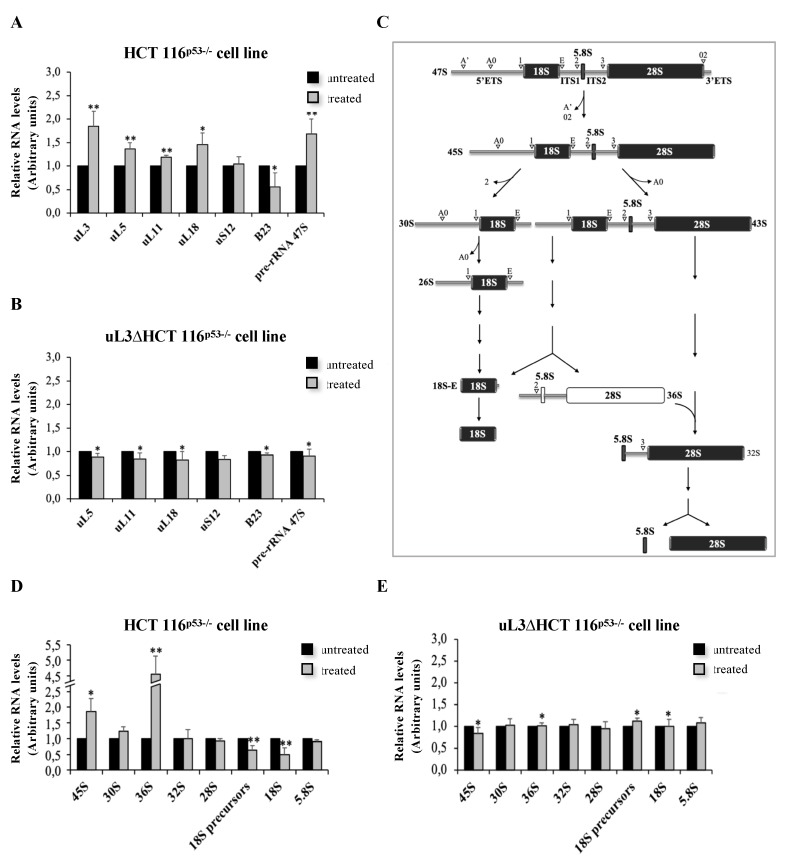
LQ1 treatment induces nucleolar stress and impairs rRNA processing. Total RNA from HCT 116^p53−/−^ (**A**) and uL3ΔHCT 116^p53−/−^ (**B**) cells, untreated or treated with 10 μM of LQ1 for 48 h, was subjected to RT–qPCR with primers specific for indicated genes (Table 2). Quantification of signals is shown. Bars represent the mean of triplicate experiments; error bars represent the standard deviation. * *p* < 0.05, ** *p* < 0.01 vs. untreated cells set at 1. (**C**) Schematic representation of 47S rRNA maturation process. Cleavage sites are indicated with white arrows. Relative expression levels of rRNA, from HCT 116^p53−/−^ (**D**) and uL3ΔHCT 116^p53−/−^ (**E**) cells, untreated or treated with 10 μM of LQ1 for 48 h analyzed by RT–qPCR with primers specific for intermediates and mature rRNAs (Table 2). Bars represent the mean of triplicate experiments; error bars represent the standard deviation. * *p* < 0.05, ** *p* < 0.01 vs. untreated cells set at 1.

As shown in Figure 3A, in the presence of uL3, the treatment of HCT 116^p53−/−^ cells with LQ1 caused a significant increase of uL3, uL5, uL11, and uL18 mRNA levels and a decrease of B23/NPM transcript levels. The observed alteration in the expression of these proteins is a hallmark of the activation of nucleolar stress [22]. When uL3 expression was switched off, LQ1 failed to exert its effects on the expression of tested ribosomal and nucleolar proteins (Figure 3B). In addition, uL5 and uL8 protein levels were analyzed by Western blotting, as shown in Figure S5A. These data indicate that LQ1 treatment caused nucleolar stress depending on uL3 status.

Within the nucleolus, ribosomal genes are transcribed by RNA polymerase I (Pol I) to produce the pre-rRNA 47S, a single transcript that is then cleaved and processed to generate the mature 28S, 18S, and 5.8S rRNAs. In this precursor, the mature rRNAs are flanked by non-coding spacer sequences, which include the 5′ and 3′ external transcribed spacers (ETS) and internal transcribed spacers (ITS) 1 and 2. These sequences contain several cleavage sites and are gradually eliminated by the sequential action of endo- and exoribonucleases schematically reported in Figure 3C [37]. In the presence of uL3, 48 h of the LQ1 treatment caused an accumulation of about 40% of the 47S pre-rRNA (Figure 3A). LQ1 failed to cause this effect when uL3 was silenced. In fact, in uL3∆HCT 116^p53-/-^ cells, the production of the 47S pre-rRNA appeared to be unchanged (Figure 3B).

In order to better characterize the role of LQ1 in pre-rRNA processing, HCT 116^p53−/−^ and uL3∆HCT 116^p53−/−^ cells were treated with LQ1 at 10 μM. Then, 48 h later, total RNA was extracted from cell lysates and the relative abundance of intermediates and mature transcripts was determined by RT–qPCR with specific primers (Table 2). The initial cleavage of the 47S pre-rRNA at sites A’ in the 5′-ETS and 02 in the 3′-ETS led to the production of 45S pre-rRNA (Figure 3C). As shown in Figure 3D, LQ1-treated HCT 116^p53−/−^ cells accummulate 45S transcript. Of interest, LQ1 treatment also resulted in a strong accumulation of 36S pre-rRNA (Figure 3D). The presence of 36S pre-rRNA that is normally expressed at low levels in normal cells is due to the inhibition of processing at Site 2 within ITS1 (Figure 3C). The accumulation of this characteristic transcript might not affect the subsequent maturation of the 5.8S and 28S rRNA [38]. In fact, we observed levels of 32S pre-rRNA, 28S, and 5.8S rRNAs comparable with untreated cells (Figure 3D). Of interest, in uL3-deleted cells, LQ1 failed to produce the accumulation of 45S and 36S rRNA precursors (Figure 3E), indicating that uL3 is crucial to activating this characteristic alternative maturation pathway that, in turn, is able to activate nucleolar stress pathway.

In addition, LQ1, in the presence of uL3, also affects the production of 30S pre-rRNA, the precursor of 18S rRNA. In fact, we observed an increase of about 30% of the 30S transcript upon exposure of cells to LQ1 (Figure 3D). The accumulation of 30S pre-rRNA was due to the inhibition of processing within 5′ETS, which caused a strong reduction (about 50%) of 18S rRNA, as shown in Figure 3D.

All these data show that the inhibition of pre-rRNA processing is a mechanism of action of LQ1.

### 3.4. Effects of LQ1 on Cell Cycle and Programmed Cell Death

**Figure 4:**
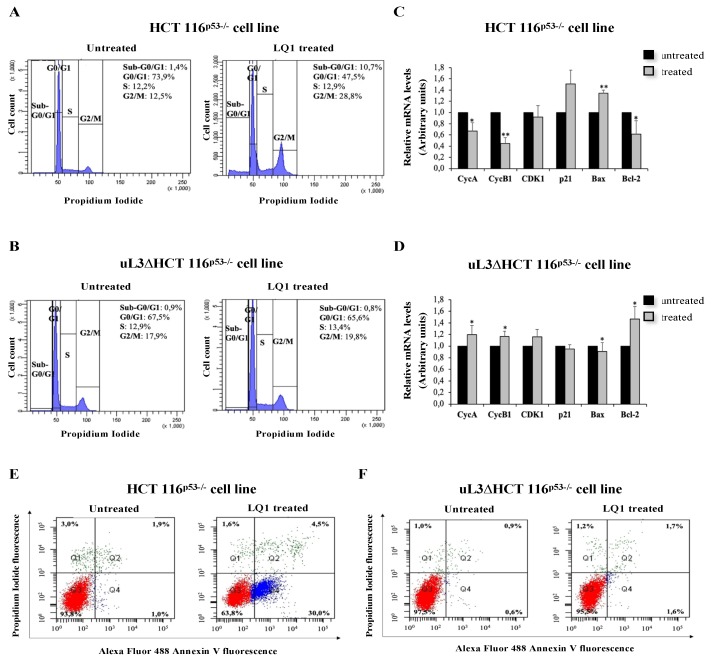
LQ1 treatment leads to cell cycle arrest and induces early apoptosis in colon cancer cells. HCT 116^p53−/−^ (**A**) and uL3ΔHCT 116^p53−/−^ (**B**) cells were incubated with 10 μM of LQ1 for 48 h, and the cell cycle distribution was evaluated using PI staining and flow cytometry analysis. Total RNA from HCT 116^p53−/−^ (**C**) and uL3ΔHCT 116^p53−/−^ (**D**) cells, untreated or treated with 10 μM of LQ1 for 48 h, was subjected to RT–qPCR with primers specific for indicated genes (Table 2). Quantification of signals is shown. Bars represent the mean of triplicate experiments; error bars represent the standard deviation. * *p* < 0.05, ** *p* < 0.01 vs. untreated cells set at 1. HCT 116^p53−/−^ (**E**) and uL3ΔHCT 116^p53−/−^ (**F**) cells were incubated with 10 μM of LQ1 for 48 h. Then, cell death was assessed by FACS analysis of Annexin V and PI staining. Representative dot plots are shown.

As already mentioned, the induction of nucleolar stress is associated with the activation of a variety of pathways that led to cell cycle arrest and/or apoptosis [21,35]. Consequently, to further understand whether the observed alterations in pre-rRNA processing were associated with induction of nucleolar stress, we performed a cell cycle analysis in the condition of uL3 silencing after exposure of cells to LQ1. To this aim, HCT 116^p53−/−^ and uL3∆HCT 116^p53−/−^ cells were treated with LQ1 at 10 μM. Then, 48 h later, cells were collected and analyzed by flow cytometry. As shown in Figure 4A, upon LQ1 treatment, the percentage of cells in the G2/M phase was increased and that in the G0/G1 phase was correspondingly decreased. Moreover, the percentage of cells in the S phase remained constant. Therefore, these results indicate that LQ1 delayed cell cycle progression by inducing G2/M arrest in HCT 116^p53−/−^ cells. Of note, cells stably silenced of uL3 resulted in insensitivity to the treatment (Figure 4B). These results suggested that LQ1 allowed HCT 116^p53−/−^ cells to produce G2/M arrest through uL3. Next, we studied the expression levels of Cyclin B1, CDK1, and Cyclin A as regulators of the G2/M transition [39]. To this aim, HCT 116^p53−/−^ and uL3∆HCT 116^p53−/−^ cells were treated with LQ1 at 10 μM for 48 h. Total RNA was extracted from cells and analyzed for the expression of cell cycle-related genes (Table 2 and Figure 4C,D). As shown in Figure 4C and Figure S5B, LQ1 caused a marked and significant downregulation of Cyclin B1 (CycB1) at mRNA and protein levels, respectively, according to G2/M arrest. In addition, the expression of G2 specific marker Cyclin A (CycA) was also reduced. These data indicate that LQ1 treatment decreases the levels of proteins involved in the entry into mitosis. When CDK1 mRNA levels were assessed, no significant alterations were observed between untreated and treated cells (Figure 3C), indicating that CDK1 was not involved in G2/M arrest caused by LQ1. To better understand the molecular mechanism by which LQ1 influences the G2/M transition, we measured p21 levels. As shown in Figure 4C and Figure S5B, an increase of p21 at mRNA and protein levels, respectively, was observed in treated cells when compared to control (untreated cells). These effects were not observed in the condition of uL3 depletion (Figure 4D).

Of interest, cell cycle analysis showed that the treatment of HCT 116^p53−/−^ cells with LQ1 associated with the presence of apoptotic cells represented by a sub G0/G1 population seen to the left of the G0/G1 peak (Figure 4A), which disappeared after uL3 deletion (Figure 4B).

As a marker of apoptosis, the ratio of the pro-apoptotic protein Bax/anti-apoptotic protein Bcl-2 was determined. Our results showed LQ1 treatment caused a significative induction of Bax at mRNA and protein levels (Figure 4C and Figure S5A), while the expression level of Bcl-2 was downregulated (Figure 4C).

When uL3 was switched off, the treatment with LQ1 was associated with a strong increase of anti-apoptotic Bcl-2 mRNA (Figure 4D), suggesting the activation of mechanisms aimed at resisting the apoptosis induced by LQ1 [40].

In order to confirm that LQ1 decreased cell survival by inducing apoptosis, we performed Annexin V-Alexa Fluor 488/PI dual staining. HCT 116^p53−/−^ and uL3ΔHCT 116^p53−/−^ cells were treated with 10 μM of LQ1 for 48 h and then evaluated with Annexin V-Alexa Fluor 488. As shown in Figure 4E, in HCT 116^p53−/−^, LQ1 treatment significantly increased the percentage of early apoptotic cells (Annexin V^+^ and PI^+^) from 1% in the untreated cells to 30% in treated cells, suggesting that apoptosis plays an important role in the antiproliferative effects of LQ1 on colon cancer cells.

Of note, in uL3ΔHCT 116^p53−/−^ cells, LQ1 treatment failed to activate apoptosis, indicating that uL3 is essential to induce LQ1–mediated apoptosis (Figure 4F). In addition, to further verify that LQ1 treatment was specifically mediated by uL3, we performed rRNA processing and cell cycle analysis in another cell line, namely, eL8ΔHCT 116^p53−/−^ cells, stably silenced of ribosomal protein eL8, arbitrarily chosen ribosomal protein, derived from parental HCT 116^p53−/−^ (Figure S6A). The results of qPCR analysis, as shown in Figure S6B, demonstrate that the treatment of these cells with LQ1 aptamer caused a significant increase of uL3, uL5, uL11, uL18 mRNA levels, and a decrease of B23/NPM transcript levels. Furthermore, an accumulation of pre-rRNA 47S after LQ1 treatment was observed. These data indicate that LQ1 induces nucleolar stress by impairing rRNA processing in eL8ΔHCT 116^p53−/−^ cells, as well as in parental cells expressing uL3 and eL8 (Figure 3). Cell cycle and apoptosis analysis in the presence of eL8 or upon eL8 silencing after exposure of cells to LQ1, as shown in Figure S7, demonstrate that LQ1 treatment caused cell cycle arrest in G2/M and apoptosis in eL8ΔHCT 116^p53−/−^ as in parental HCT 116^p53−/−^ cells.

All these data indicate that LQ1 activity on the progression of cell cycles and on apoptosis depends on uL3 status.

## 4. Discussion

G4-aptamers have been extensively documented as therapeutic agents able to activate potent antiproliferative effects in a variety of cancer cell lines [7]. Among G4-aptamers, the anticancer AS1411 is the only one for which Phase II has been completed [41]. AS1411 has been the topic of a considerable number of investigations concerning both its chemico-physical characteristics and mechanism of action that indicated as target the nucleolin, which is a multifunctional protein overexpressed in cytoplasms and on cell surfaces of many tumor types [42]. Previously, the physical and antiproliferative properties of other G-quadruplex-forming sequences were described (including ODN GRO29A, which is the precursor of AS1411) and the cancer-selective antiproliferative activity was suggested to be a general characteristic of G-rich ODNs sequences, potentially forming G-quadruplex structures [4]. To date, one of the major challenges in the field of G4-aptamers as anticancer agents is to better understand the mechanisms by which these molecules target and kill cancer cells. Herein, we have investigated the structural characteristic and cytotoxic activities of two TBA derivatives, namely, LQ2 and LQ3, and we have identified the molecular mechanism underlying antiproliferative response of HCT 116^p53−/−^ and uL3ΔHCT 116^p53−/−^ cells to LQ1. LQ1 is a previously investigated TBA derivative [11] composed of L-nucleosides except for those in the small loops. LQ2 and LQ3 differ from LQ1 only by the D/L-residues composition of the small loops. All the investigated ODNs adopt chair-like left-handed G-quadruplex structures. Results of MTT assays, reported in this paper, demonstrated a low cytotoxic activity of LQ2 and LQ3 on HCT 116^p53−/−^ and uL3ΔHCT 116^p53−/−^ cells compared to that of LQ1 on HCT 116^p53−/−^ and TBA on both cell lines in the same experimental conditions. These results clearly indicate a key role of the small loops in the biological activity of this family of TBA derivatives. Furthermore, results from an MTT assay on HCT 116^p53−/−^ and uL3ΔHCT 116^p53−/−^ cells treated with LQ1 and L-TBA strongly suggested that different pathways were involved in the cytotoxic activities of these two TBA derivatives. In particular, the results of our studies demonstrate that LQ1 is able to activate a novel p53-independent and uL3 dependent nucleolar stress pathway. The nucleolus is the site of ribosome biogenesis; however, recently, works have suggested a new non-canonical role for the nucleolus in regulating important cellular events correlate to cancer development and progression such as cell cycle, cellular senescence, apoptosis, genome repair, and stability [43,44]. Perturbation of the nucleolar function and/or structure with consequent induction of nucleolar stress activates different signaling pathways, dependent or not of p53, resulting in cell cycle arrest and/or apoptosis [21,45].

In an effort to identify the molecular mechanism underlying the cytotoxic activity of LQ1, we became interested in evaluating its effects on nucleolar function. Alterations of the expression profile of a specific subset of r-proteins, including uL3, uL5, and uL18, and of the nucleolar marker B23/NPM in HCT 116^p53−/−^ cells treated with LQ1, pointed to nucleolar stress as the pathway activated by this G-4 aptamer. Furthermore, analysis of the rRNA processing pathway demonstrated that the treatment of cells expressing uL3 with LQ1 leads to alteration in the processing of 47S pre-rRNA with consequent accumulation of 45S, 36S, and 30S rRNA precursors. All these data suggest that the treatment of cells with LQ1 impairs ribosomal biogenesis via inhibition of pre-rRNA processing, with consequent activation of the nucleolar stress response and stabilization of a subset of ribosomal proteins that are the specific hallmark of the nucleolar stress condition, including uL3, uL5, and uL18 [21,45]. The absence of these effects in uL3 deficient cells (uL3ΔHCT 116^p53−/−^ cells) clearly demonstrated that LQ1 is able to trigger a p53-independent but uL3-dependent nucleolar stress pathway. Altogether, these results have revealed a previously unknown role of uL3 in the mechanism of action of LQ1; in fact, the expression of uL3 is activated by LQ1 treatment in HCT 116^p53−/−^ cells and the silencing of uL3 in uL3ΔHCT 116^p53−/−^ cells completely abolish the cytotoxic effect of LQ1.

It is known that cell cycle arrest and/or apoptosis are the common cell responses to the activation of nucleolar stress [21]. Here, we have shown that, in the presence of uL3, LQ1 treatment causes cell cycle arrest in G2/M and induces early apoptosis. These effects are associated with a strong reduction of proteins regulating the cell cycle progression as Cyclin B1 and Cyclin A [46] and a related upregulation of uL3 and p21 expression. All these effects are not present in cell line stable silenced for uL3 expression, confirming a key role of this protein in the biological activity of LQ1. On the basis of our results, we speculate that LQ1 treatment causes the release of uL3, as ribosome free protein, from the nucleolus to the nucleoplasm, where it positively regulates p21 expression. It has been demonstrated that p21 inhibits mitosis through the degradation of the mitotic cyclins contributing to G2 arrest [47,48]. Thus, we hypothesize that high p21 intracellular levels can downregulate Cyclin B1 that, in turn, as a master mitotic regulator, contribute to establishing G2/M arrest of the cell cycle. These data represent the first evidence of a connection between the antiproliferative ability of G4-aptamers and perturbation of the nucleolar function associated with the activation of the nucleolar stress pathway.

Recent data indicate that cancer cells exhibit upregulation of ribosome biogenesis and result in a high sensitivity to drugs that activate the nucleolar stress response [22]. In this context, our results may have a significant value in the development of new targeted anticancer therapies for colorectal tumors lacking p53, which are often resistant to current therapies and have a poor prognosis.

## 5. Conclusions

Our study has revealed the mechanism of action of the G4-aptamer LQ1, identifying the uL3-mediated nucleolar stress response pathway as the molecular mechanism selectively activated in colon cancer cell response. These, to date, are of particular importance, considering that, to our knowledge among G4-aptamers, AS1411 is the only one for which the mechanism of action has been elucidated so far [42].

Further research on the molecular mechanisms involved in LQ1 activity may lead to the discovery of novel therapeutic approaches for targeting cancer cells.

## Figures and Tables

**Table 1:** Sequences investigated and their melting temperatures (T_m_).

Name	Sequence ^[**a**]^	T_m_ (°C)
TBA	5′-GGTTGGTGTGGTTGG-3′	50 ^[**b**]^
L-TBA	5′-ggttggtgtggttgg-3′	50 ^[**b**]^
LQ1	5′-ggTTggtgtggTTgg-3′	52 ^[**b**]^
LQ2	5′-ggTtgggtgtggTtgg-3′	49
LQ3	5′-ggtTgggtgtggtTgg-3′	49

[**a**] D and L residues are indicated in upper and lower case, respectively. The residues in the small loops are highlighted in red. [**b**] Data from reference 17.

**Table 2:** Sequence of oligonucleotides used in RT–qPCR analysis.

Gene	Sequence
CDK1	Forward: 5′ – CATGGCTACCACTTGACCTGT – 3′ Reverse: 5′ – AAGCCGGGATCTACCATACC – 3′
CycA	Forward: 5′ – TTCATTTAGCACTCTACACAGTCACGG – 3′ Reverse: 5′ – TTGAGGTAGGTCTGGTGAAGGTCC – 3′
CycB	Forward: 5′ – CAGTCAGACCAAAATACCTACTGGGT – 3′ Reverse: 5′ – ACACCAACCAGCTGCAGCATCTTCTT – 3′
Bax	Forward: 5′ – CCCGAGAGGTCTTTTCCGAG – 3′ Reverse: 5′ – CCAGCCCATGATGGTTCTGAT – 3′
B23/NPM	Forward: 5′ – AGAAAAAGCGCCAGTGAAGA – 3′ Reverse: 5′ – TGGTGTT GATGATTGGTTTTGA – 3′
β-actin	Forward: 5′ – CCAACCGCGAGAAGATGA – 3′ Reverse: 5′ – CCAGAGGCGTACAGGGATAG – 3′
Bcl-2	Forward: 5′ – ATGTGTGTGGAGAGCGTCAACC – 3′ Reverse: 5′ – GCATCCCAGCCTCCGTTATC – 3′
p21	Forward: 5′ – CCTCAAATCGTCCAGCGACCTT – 3′ Reverse: 5′ – CATTGTGGGAGGAGCTGTGAAA – 3′
uL3	Forward: 5′ – CAAAGGCTACAAAGGGGT – 3′ Reverse: 5′ – CTCAGTGCGGTGATGGTAG – 3′
uL5	Forward: 5′ – GGGATCCAGGAACACATCGA – 3′ Reverse: 5′ – AGAAGTCCAGGCCGTAGATACCA – 3′
uL11	Forward: 5′ – AGTCGTATACCTGAGGTGCACCGGA – 3′ Reverse: 5′ – GCCATCAACATTACAGCCCACTGAC – 3′
uL18	Forward: 5′ – TGGAACCGTCCCAAAATGTC – 3′ Reverse: 5′ – GAGGAAGCTTGCCTTCTTTTGAG – 3′
uS12	Forward: 5′ – CGAGACCAGAAGTGGCATGA – 3′ Reverse: 5′ – GCATGAGAAGCACCTCCAAAAG – 3′
47S	Forward: 5′ – GCTGACACGCTGTCCTCTG – 3′ Reverse: 5′ – ACGCGCGAGAGAACAGCAG – 3′
45S	Forward: 5′ – GCCTTCTCTAGCGATCTGAGAG – 3′ Reverse: 5′ – CCATAACGGAGGCAGAGACA – 3′
36S	Forward: 5′ – GCGGAGGTTTAAAGACCC – 3′ Reverse: 5′ – CCAGACGAGACAGCAAAC – 3′
32S	Forward: 5′ – GTCAGCGGAGGAGAAGAA – 3′ Reverse: 5′ – CTCGATCAGAAGGACTTGG – 3′
30S	Forward: 5′ – CCTCTGACGCGGCAGACAGC – 3′ Reverse: 5′ – CTCCAGGAGCACCGCAAGGG – 3′
18S precursors	Forward: 5′ – GTTCAAAGCAGGCCCGAGCC – 3′ Reverse: 5′ – AGCGGCGCAATACGAATGCC – 3′
28S	Forward: 5′ – CAGGGGAATCCGACTGTTTA – 3′ Reverse: 5′ – ATGACGAGGCATTTGGCTAC – 3′
18S	Forward: 5′ – AAACGGCTACCACATCCAAG – 3′ Reverse: 5′ – CCTCCAATGGATCCTCGTTA – 3′
5.8S	Forward: 5′ – CTCTTAGCGGTGGATCACTC – 3′ Reverse: 5′ – GACGCTCAGACAGGCGTAG – 3′

## Supplementary Materials

The following are available online at https://www.mdpi.com/2218-273X/10/4/583/s1, Figure S1: CD heating and cooling experiments. Figure S2: Two-dimensional NMR experiments. Figure S3: Two-dimensional NMR experiments. Figure S4: Representative western blotting analysis of uL3 in HCT 116^p53−/−^ and uL3ΔHCT 116^p53−/−^ cells. Figure S5: LQ1 treatment modulates the expression of nucleolar stress, cell cycle- and apoptosis-related genes. Figure S6: LQ1 treatment induces nucleolar stress in eL8ΔHCT 116^p53−/−^ cells. Figure S7: LQ1 treatment causes cell cycle arrest and apoptosis in eL8ΔHCT 116^p53−/−^ cells. Figure S8: Full-length blots of Western blotting showed in Figure S4. Figure S9: Full-length blots of Western blotting showed in Figure S5A. Figure S10: Full-length blots of Western blotting showed in Figure S5B. Figure S11: Full-length blots of Western blotting showed in Figure S6A. Table S1: Chemical shift values.
