# Supplementary material for: uL3 Mediated Nucleolar Stress Pathway as a New Mechanism of Action of Antiproliferative G-quadruplex TBA Derivatives in Colon Cancer Cells
Source: Biomolecules. 2020 Apr 10;10(4):583. doi: 10.3390/biom10040583 (PMC7226491; doi:10.3390/biom10040583)
Supplement: Supplementary file 1 [file biomolecules-10-00583-s001.pdf]

**uL3 mediated nucleolar stress pathway as a new mechanism of action of antiproliferative G-quadruplex TBA derivatives in colon cancer cells**

Annalisa Pecoraro[1], Antonella Virgilio[1], Veronica Esposito[1], Aldo Galeone[1], Giulia Russo[1]*, Annapina Russo[1]*.

[1]Department of Pharmacy, University of Naples “Federico II”, Via Domenico Montesano 49, 80131 Naples, Italy

*Correspondence to: Annapina Russo and Giulia Russo, Department of Pharmacy, University of Naples “Federico II”, Via Domenico Montesano 49, 80131 Naples, Italy, Tel: +39 081 678414; +39 081 7463074; Email: annapina.russo@unina.it; giulia.russo@unina.it

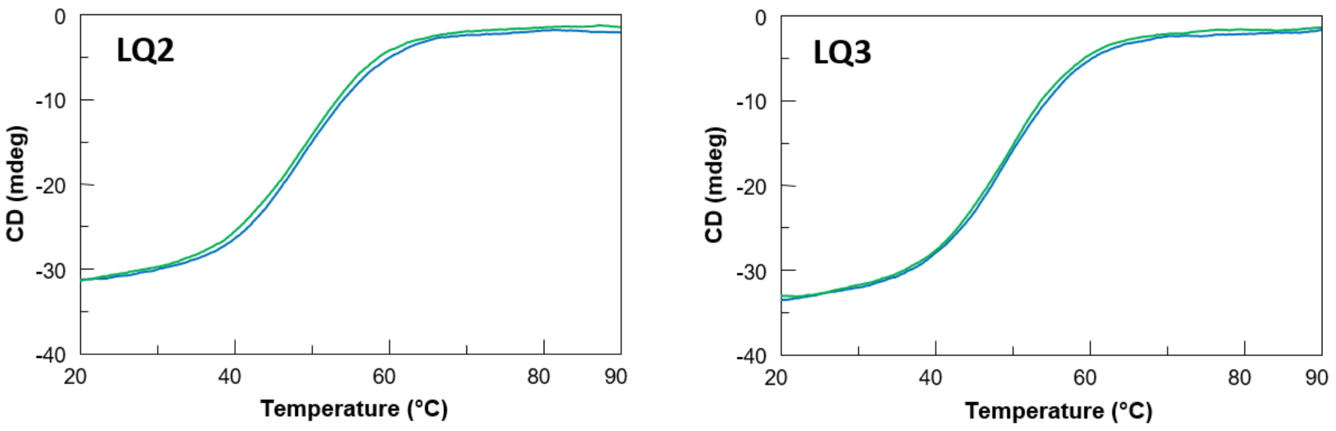


**Figure S1**. CD heating and cooling experiments. CD melting (blue) and annealing (green) curves registered as a function of temperature of the modified quadruplexes LQ2 and LQ3 at 294 nm. CD data were recorded in a 0.1 cm pathlength cuvette with a scan rate of 30°C/h at 50μM ODN strand concentration in $K^+$ buffer (10 mM $KH_2PO_4$/$K_2HPO_4$, 70 mM KCl, pH 7.0).

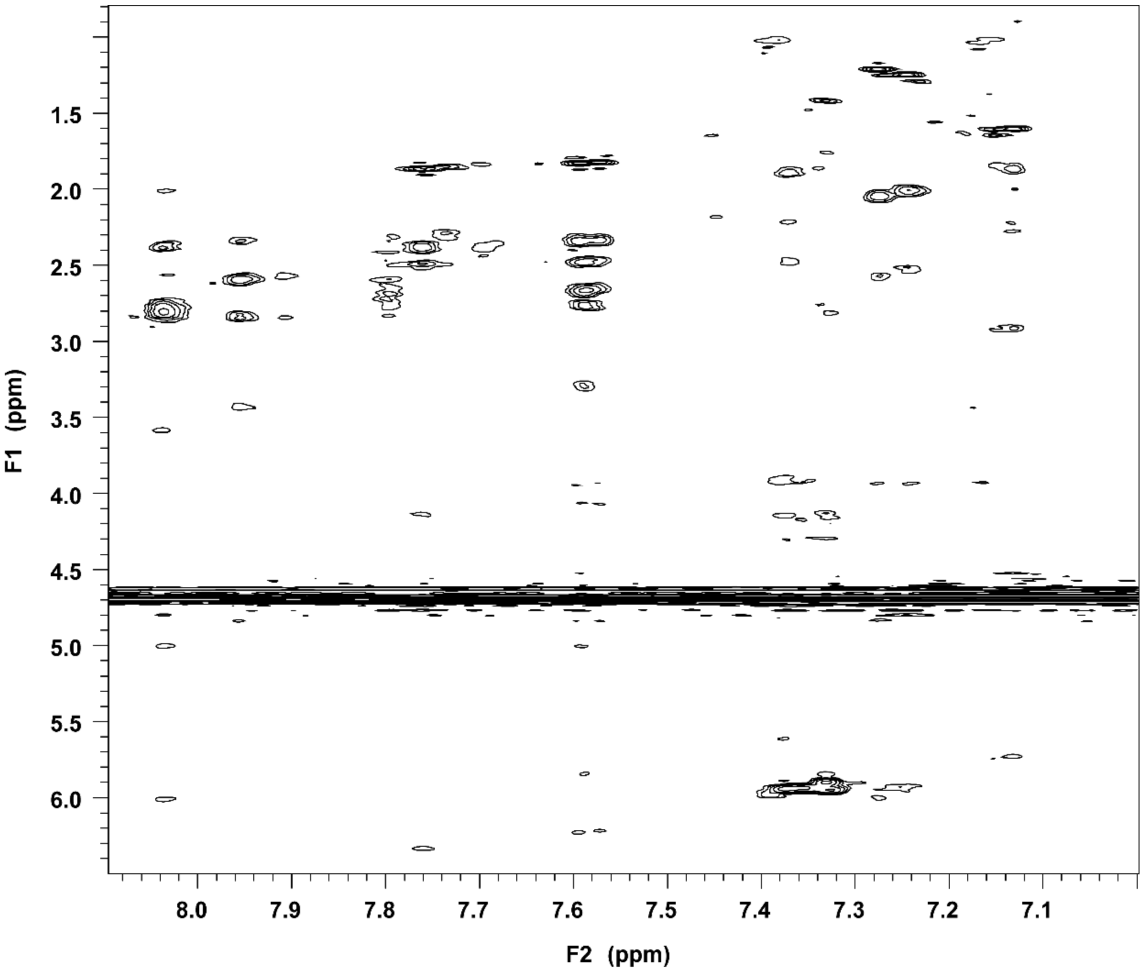


**Figure S2**. Two-dimensional NMR experiments. Expanded 2D NOESY spectrum of LQ2 (500 MHz; 25°C; strand concentration 3 mM; 10 mM $KH_2PO_4/K_2HPO_4$, 70 mM KCl and 0.2 mM EDTA, pH 7.0 in H2O/D2O 9:1; total volume 0.6 ml; mixing time 180 ms) correlating aromatic base protons and sugar protons.

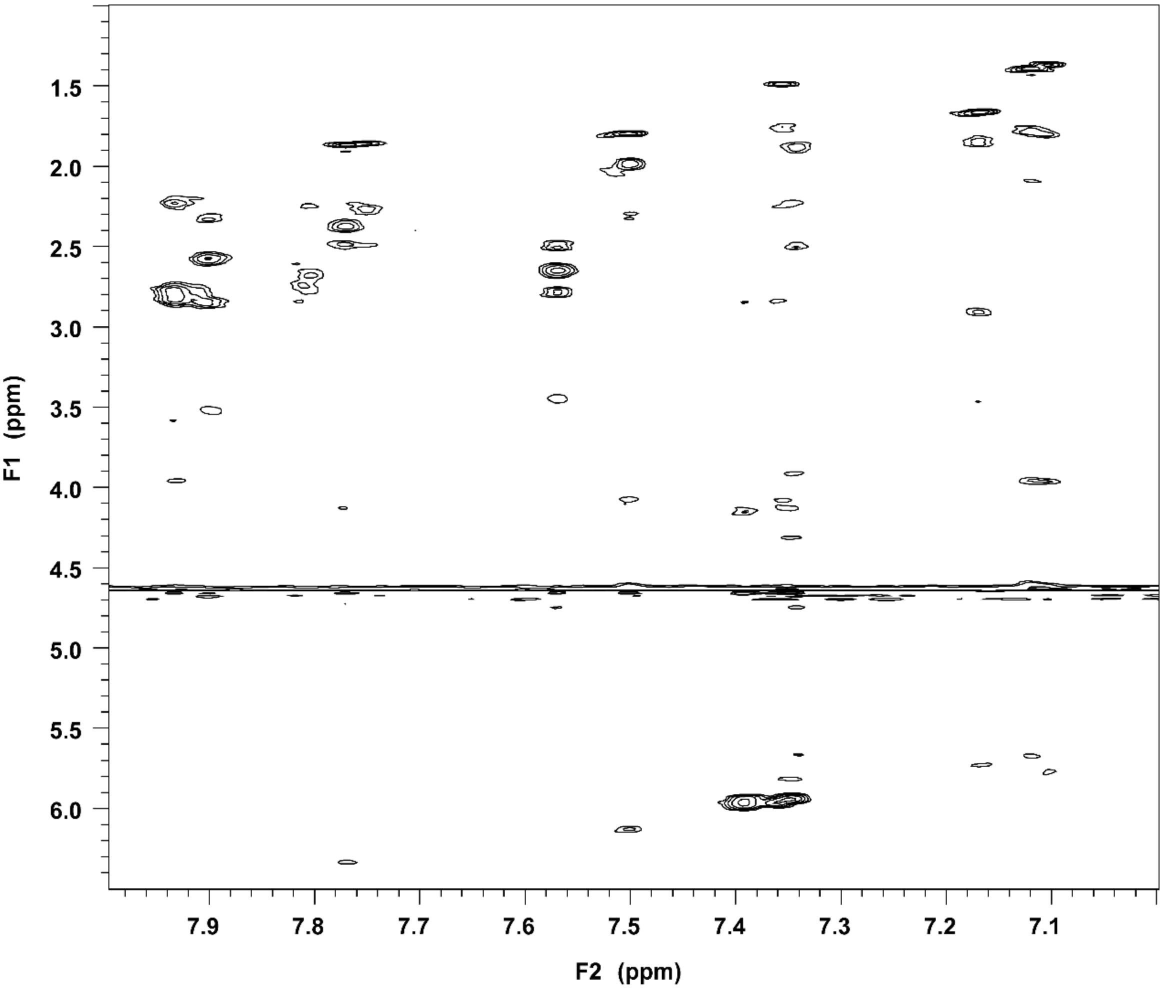


**Figure S3**. Two-dimensional NMR experiments. Expanded 2D NOESY spectrum of LQ3 (500 MHz; 25°C; strand concentration 3 mM; 10 mM $KH_2PO_4$/$K_2HPO_4$, 70 mM KCl and 0.2 mM EDTA, pH 7.0 in $H_2O$/$D_2O$ 9:1; total volume 0.6 ml; mixing time 180 ms) correlating aromatic base protons and sugar protons.

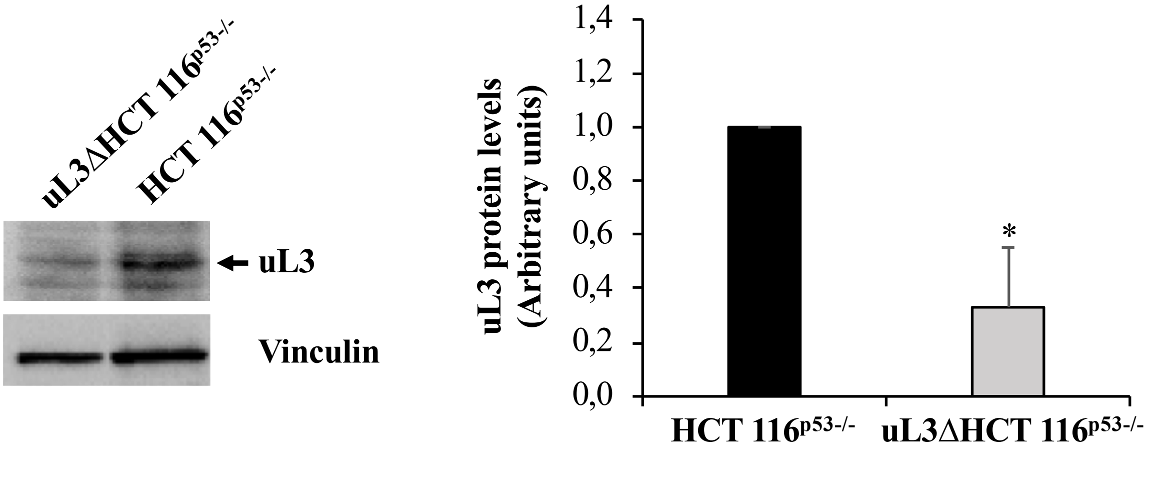


**Figure S4**. Representative western blotting analysis of uL3 in HCT $116^{p53-/-}$ and uL3ΔHCT $116^{p53-/-}$ cells. Protein extracts were analyzed by western blotting with the indicated antibodies. Vinculin was used as loading controls. Full-length blot is presented in Figure S8. Quantification of signals is shown. Bars represent the mean of triplicate experiments; error bars represent the standard deviation. *$p < 0.05$ vs. HCT $116^{p53-/-}$ cells set at 1.

A

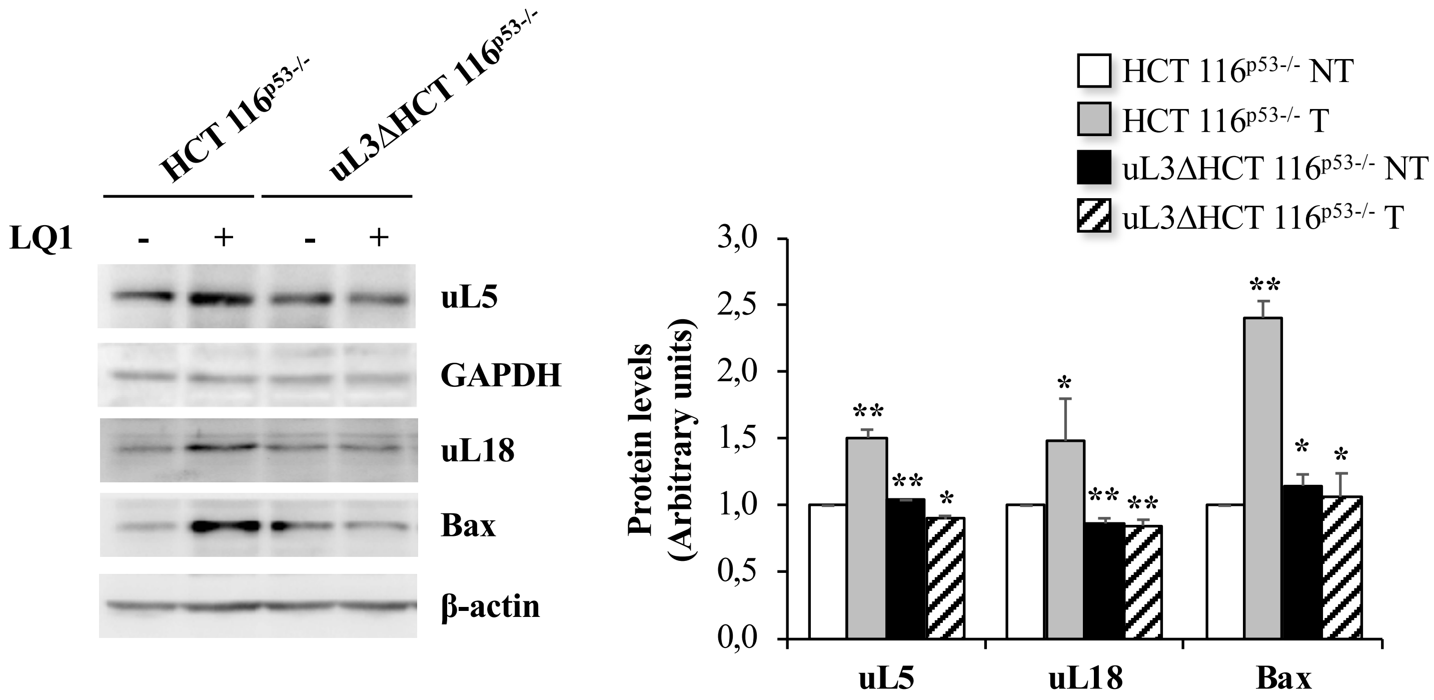


B

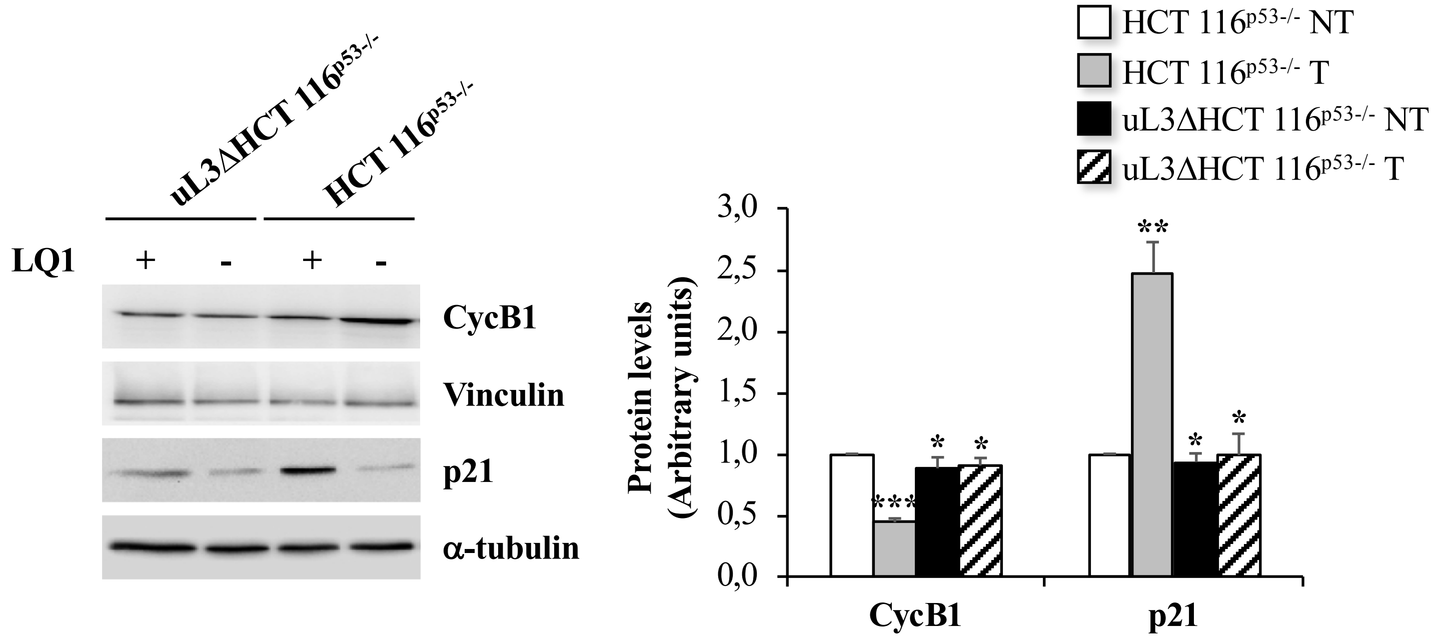


**Figure S5**. LQ1 treatment modulates the expression of nucleolar stress, cell cycle and apoptosis related genes. Representative western blot analysis of uL5, uL18 and Bax (**A**), CycB1 and p21 (**B**). HCT 116$^{p53-/-}$ and uL3ΔHCT 116$^{p53-/-}$ cells were treated with 10 μM of LQ1 for 48h. After treatment, protein extracts from the samples were analyzed by western blotting with antibodies against indicated proteins. GAPDH, β-actin, Vinculin and α-tubulin ware used as loading controls. Full-length blot is presented in Figures S9 and S10. Quantification of signals is shown. Bars represent the mean of triplicate experiments; error bars represent the standard deviation. *P<0.05, **P<0.01, ***P<0.001 vs. HCT 116$^{p53-/-}$ cells set at 1. NT: untreated; T: LQ1 treated.

**A**

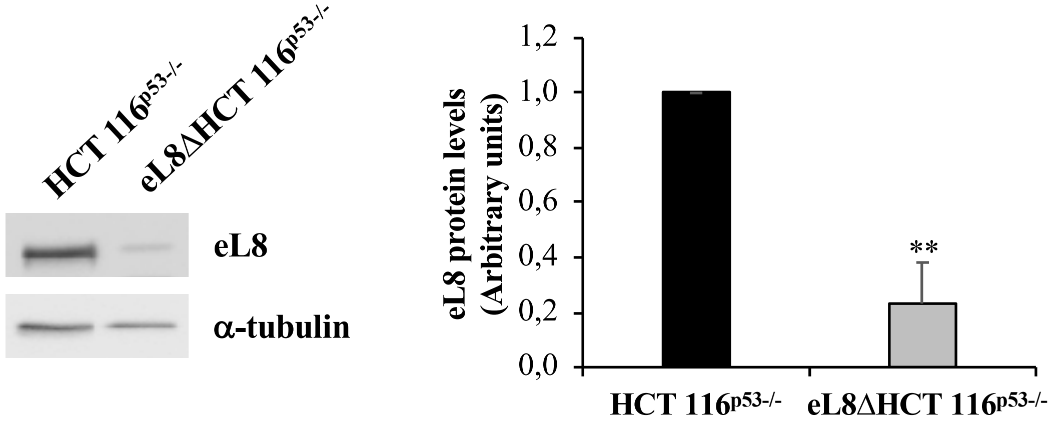


**B**

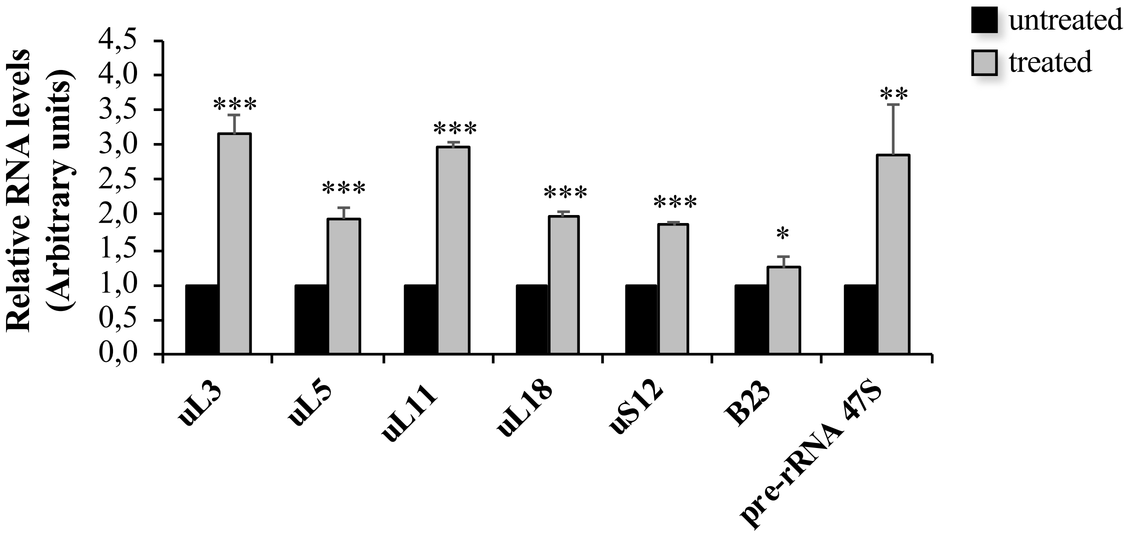


**Figure S6**. LQ1 treatment induces nucleolar stress in eL8ΔHCT 116$^{p53-/-}$ cells. (**A**) Representative western blot analysis of eL8 in HCT 116$^{p53-/-}$ and eL8ΔHCT 116$^{p53-/-}$ cells. Protein extracts were analyzed by western blotting with the indicated antibodies. **α**-tubulin was used as loading controls. Full-length blot is presented in Figure S11. Quantification of signals is shown. Bars represent the mean of triplicate experiments; error bars represent the standard deviation. **p < 0.01 vs. HCT 116$^{p53-/-}$ cells set at 1. (**B**) Total RNA from eL8ΔHCT 116$^{p53-/-}$ cells, untreated or treated with 10 μM of LQ1 for 48 h, was subjected to RT-qPCR with primers specific for indicated genes (Table 2). Quantification of signals is shown. Bars represent the mean of triplicate experiments; error bars represent the standard deviation. *P<0.05, **P<0.01, ***P<0.001 vs. untreated cells set at 1.

A

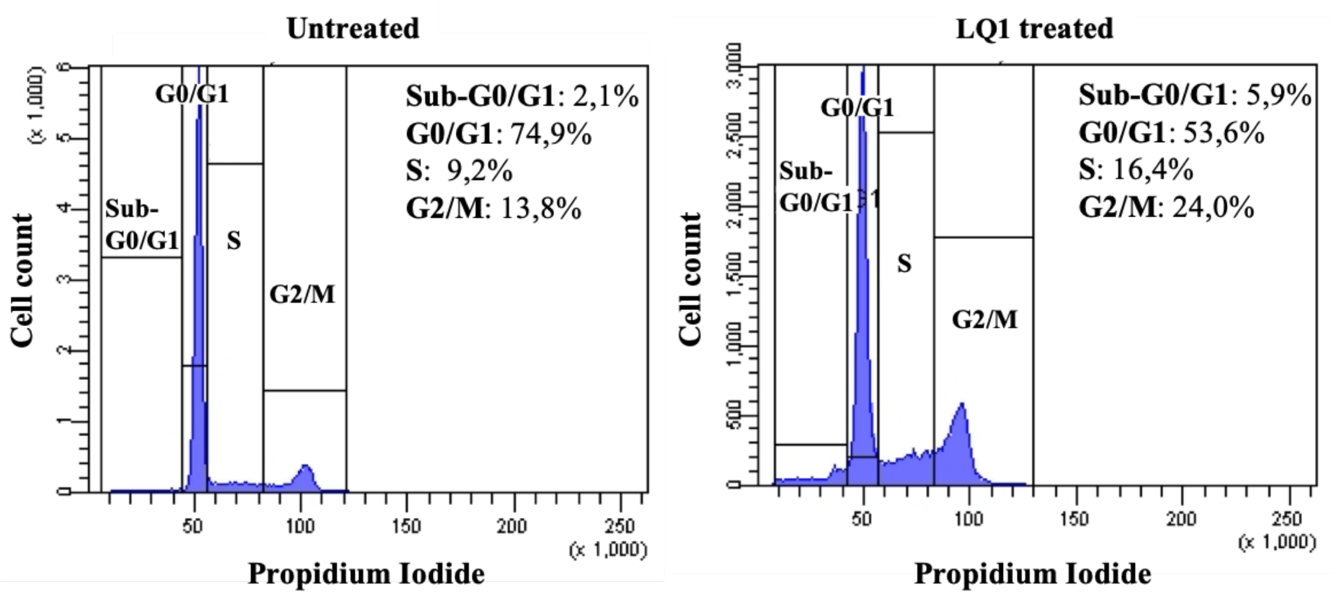


B

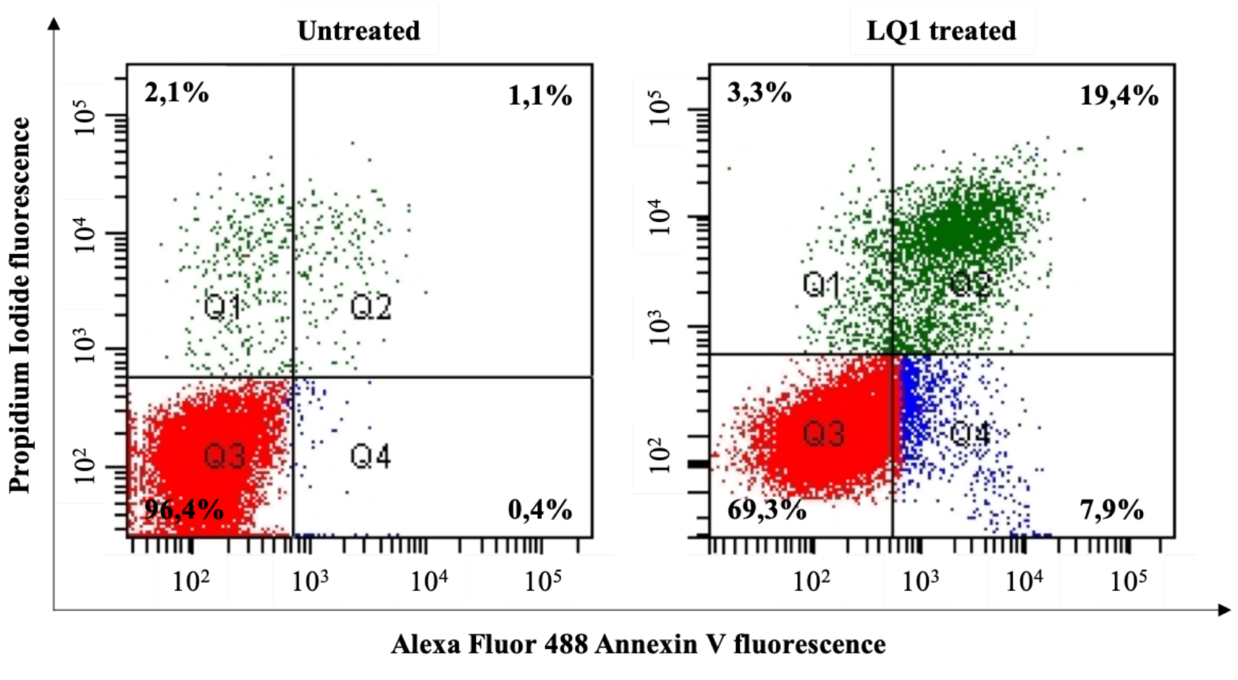


**Figure S7**. LQ1 treatment causes cell cycle arrest and apoptosis in eL8ΔHCT 116$^{p53-/-}$ cells. (**A**) Cells were incubated with 10 µM of LQ1 for 48 h and the cell cycle distribution was evaluated using PI staining and flow cytometry analysis. (**B**) Cell death was assessed by FACS analysis of Annexin V and PI staining. Representative dot plots are shown.

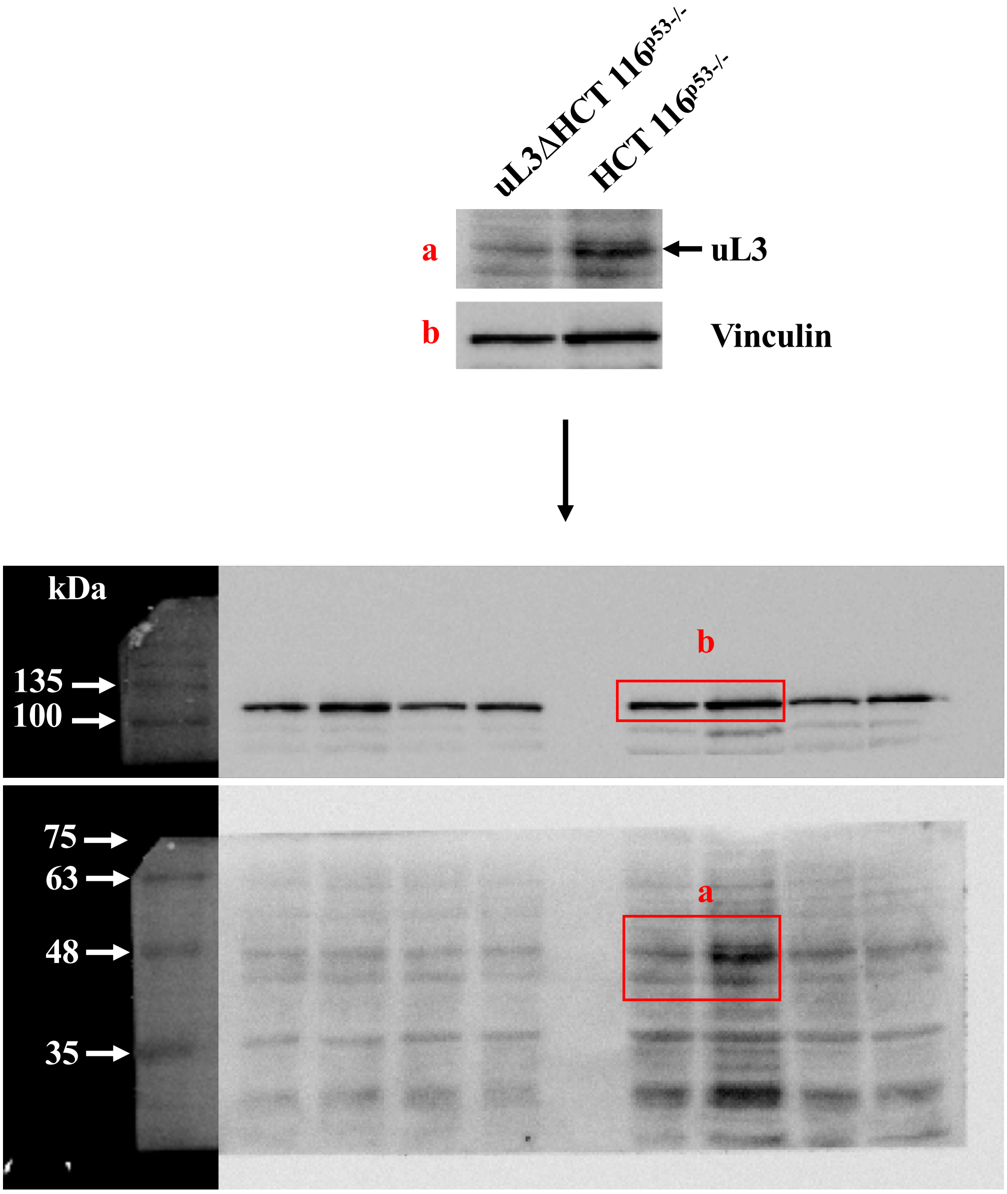


**Figure S8**. Full-length blots of western blotting showed in Figure S4.

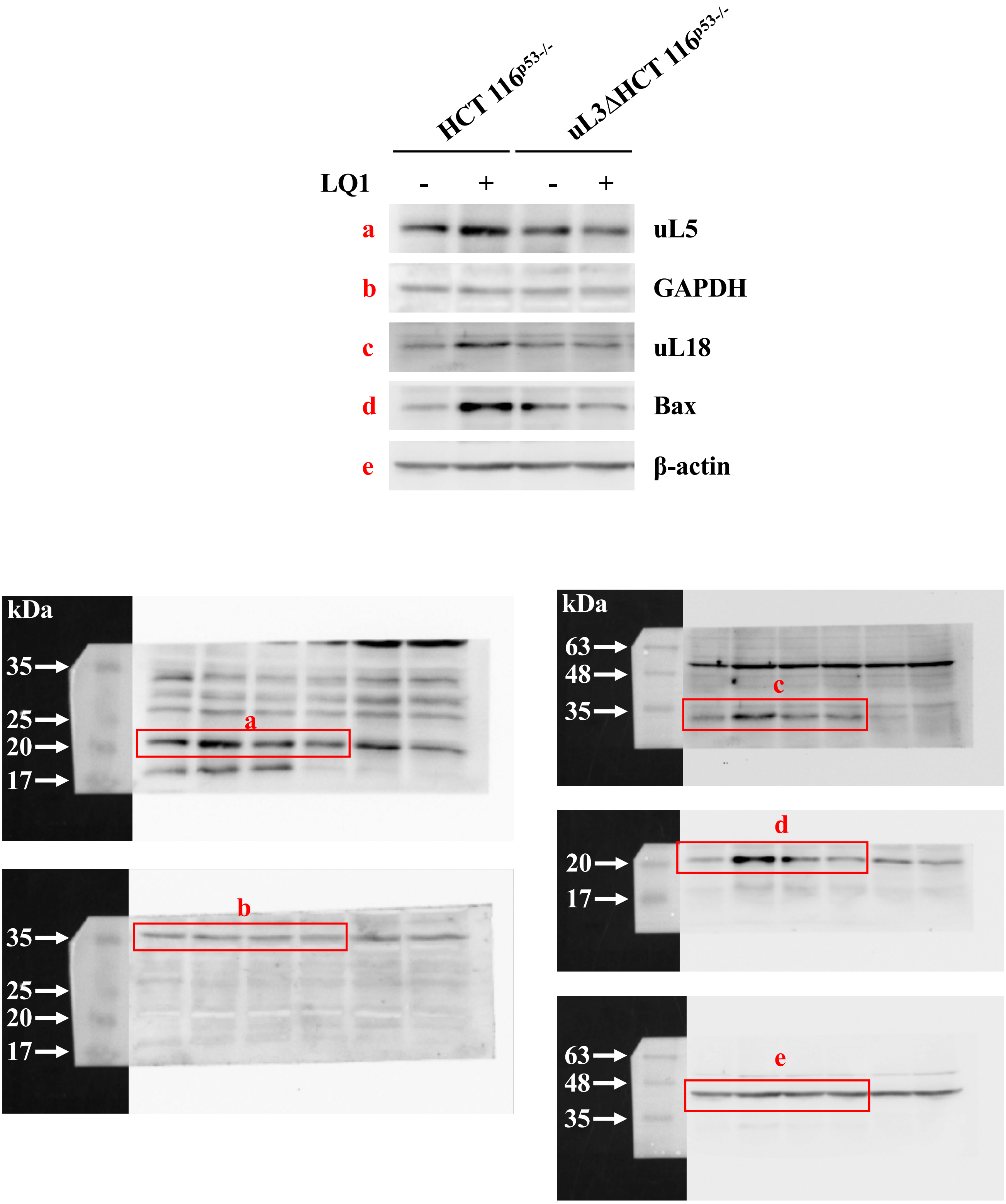


**Figure S9**. Full-length blots of western blotting showed in Figure S5A.

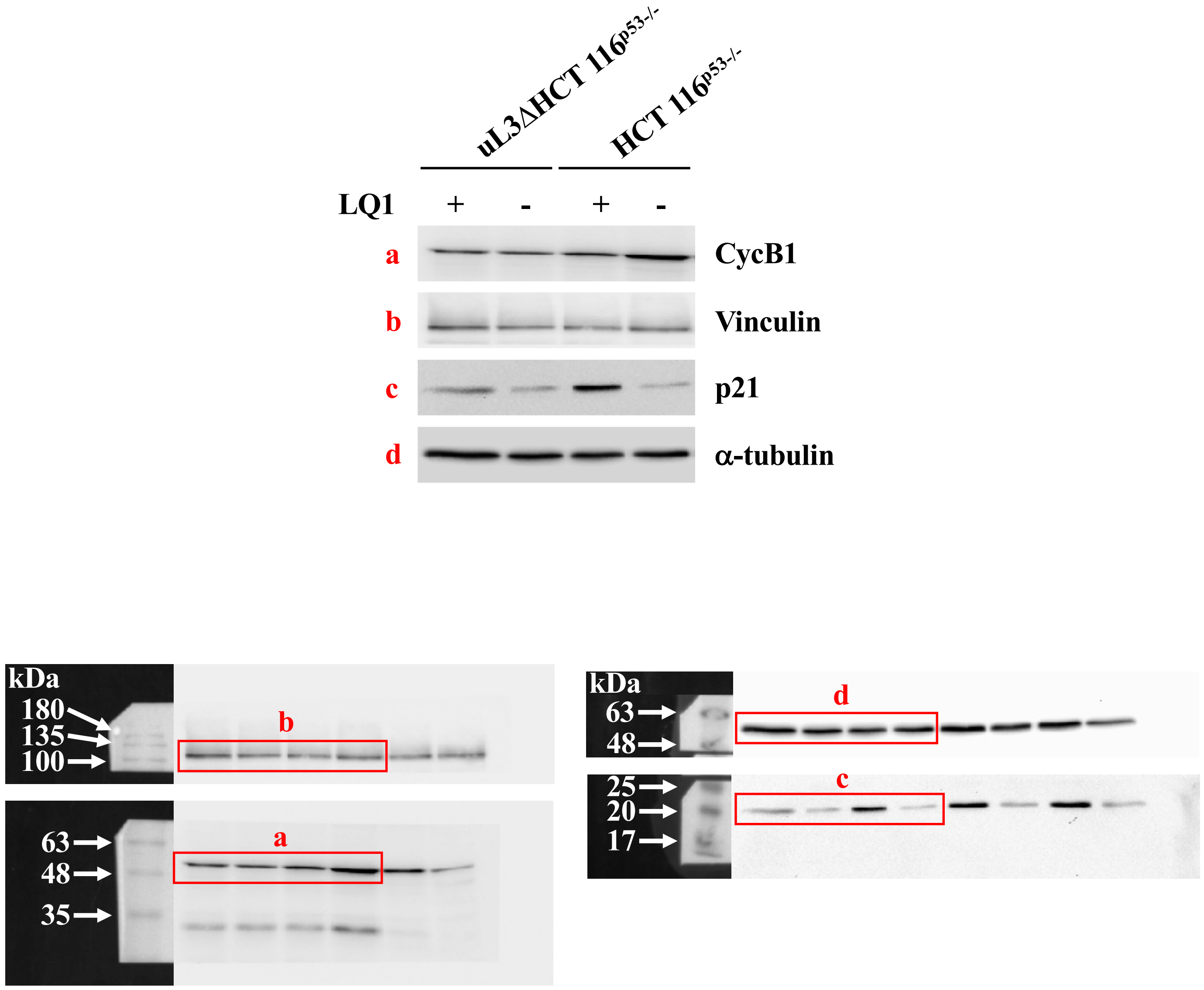


**Figure S10**. Full-length blots of western blotting showed in Figure S5B.

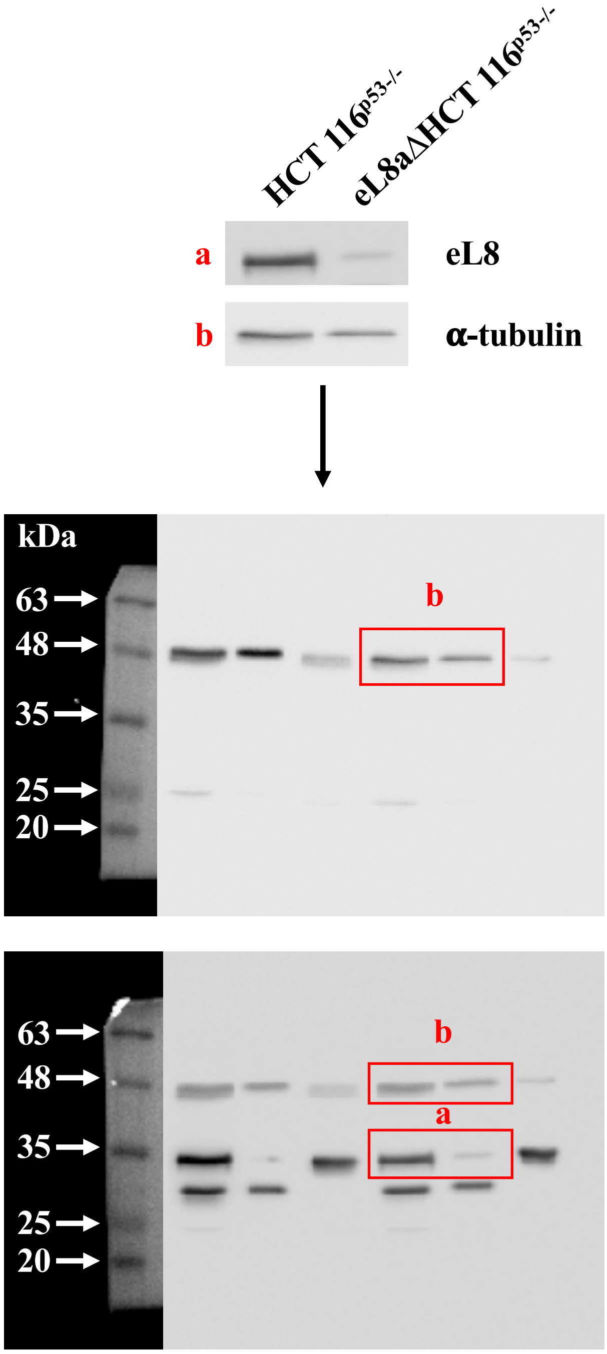


**Figure S11**. Full-length blots of western blotting showed in Figure S6A.

| **LQ2** 5'-$G_{L1}G_{L2}T_{D3}T_{L4}G_{L5}G_{L6}T_{L7}G_{L8}T_{L9}G_{L10}G_{L11}T_{D12}T_{L13}G_{L14}G_{L15}$-3' | | | | | | | |
|---|---|---|---|---|---|---|---|
| | H8/H6 | H1' | H2'/H2" | H3' | H4' | H5'/H5" | $CH_3$ |
| $\underline{G}_{L1}$ | 7.32 | 5.95 | 2.82 | 4.89 | 4.30 | 4.16 | - |
| $G_{L2}$ | 8.04 | 6.00 | 2.40/2.82 | 5.00 | 4.21 | 4.17 | - |
| $T_{D3}$ | 7.60 | 6.23 | 2.34/2.49 | 4.84 | 4.24 | 4.07 | 1.83 |
| $T_{L4}$ | 7.25 | 5.93 | 2.02/2.53 | 4.79 | 4.22 | 3.93 | 1.26 |
| $\underline{G}_{L5}$ | 7.33 | 5.88 | 2.78/3.30 | 4.77 | 4.52 | 4.06 | - |
| $G_{L6}$ | 7.59 | 5.84 | 2.49/2.68 | 5.01 | N.D. | 4.08/3.95 | - |
| $T_{L7}$ | 7.76 | 6.34 | 2.38/2.51 | 4.75 | 4.31 | 4.15 | 1.86 |
| $G_{L8}$ | 7.37 | 5.61 | 1.90/2.22 | 4.57 | 4.30 | 3.90 | - |
| $T_{L9}$ | 7.14 | 5.74 | 1.87/2.28 | 4.53 | 4.20 | N.D. | 1.60 |
| $\underline{G}_{L10}$ | 7.36 | 5.94 | 2.82/3.58 | 4.80 | 4.16 | N.D. | - |
| $G_{L11}$ | 8.04 | 6.03 | 2.38/2.78 | 5.03 | N.D. | 4.14 | - |
| $T_{D12}$ | 7.57 | 6.22 | 2.34/2.49 | 4.84 | 4.24 | 4.07/3.93 | 1.82 |
| $T_{L13}$ | 7.28 | 6.00 | 2.06/2.58 | 4.80 | 4.20 | 3.95 | 1.21 |
| $\underline{G}_{L14}$ | 7.37 | 5.94 | 2.84/3.43 | 4.84 | N.D. | N.D. | - |
| $G_{L15}$ | 7.95 | 6.05 | 2.35/2.61 | 4.80 | 4.29 | 4.16 | - |

| **LQ3** 5'-$G_{L1}G_{L2}T_{L3}T_{D4}G_{L5}G_{L6}T_{L7}G_{L8}T_{L9}G_{L10}G_{L11}T_{L12}T_{D13}G_{L14}G_{L15}$-3' | | | | | | | |
|---|---|---|---|---|---|---|---|
| | H8/H6 | H1' | H2'/H2" | H3' | H4' | H5'/H5" | $CH_3$ |
| $\underline{G}_{L1}$ | 7.36 | 5.97 | 2.84 | 4.88 | N.D. | N.D. | - |
| $G_{L2}$ | 7.81 | 5.85 | 2.25/2.68 | 4.97 | 4.61 | 4.29 | - |
| $T_{L3}$ | 7.36 | 5.85 | 2.10/2.24 | 4.86 | N.D. | N.D. | 1.49 |
| $T_{D4}$ | 7.10 | 5.78 | 1.82/2.78 | 5.00 | 4.33 | N.D. | 1.36 |
| $\underline{G}_{L5}$ | 7.35 | 5.94 | 2.80/3.45 | 4.78 | 4.30 | N.D. | - |
| $G_{L6}$ | 7.57 | 5.82 | 2.50/2.66 | 5.00 | 4.36 | 4.24 | - |
| $T_{L7}$ | 7.77 | 6.34 | 2.38/2.50 | 4.75 | 4.31 | 4.14 | 1.86 |
| $G_{L8}$ | 7.34 | 5.68 | 1.88/2.23 | 4.69 | N.D. | N.D. | - |
| $T_{L9}$ | 7.17 | 5.74 | 1.84/2.29 | 4.53 | N.D. | 3.65 | 1.66 |
| $\underline{G}_{L10}$ | 7.39 | 5.94 | 2.85/3.60 | 4.80 | 4.29 | N.D. | - |
| $G_{L11}$ | 7.93 | 5.80 | 2.23/2.80 | 5.00 | 4.25 | 4.13 | - |
| $T_{L12}$ | 7.50 | 6.13 | 2.00/2.31 | 4.62 | 4.26 | 4.08 | 1.80 |
| $T_{D13}$ | 7.12 | 5.68 | 1.78/2.08 | 4.61 | 4.28 | N.D. | 1.39 |
| $\underline{G}_{L14}$ | 7.39 | 5.96 | 2.85/3.52 | 4.80 | 4.33 | 4.18 | - |
| $G_{L15}$ | 7.90 | 6.02 | 2.34/2.59 | 4.69 | 4.12 | N.D. | - |

**Table S1.** Chemical shift values. Proton chemical shifts assignment for G-quadruplex formed by ODNs LQ2 and LQ3 (500 MHz, T = 25°C) in 10 mM KH2PO4/K2HPO4, 70 mM KCl and 0.2 mM EDTA (pH 7.0). N.D. = not determined. Residues adopting the syn glycosidic conformation are underlined.
